# Correlation in Expression between LTR Retrotransposons and Potential Host *Cis*-Targets during Infection of Antherea pernyi with ApNPV Baculovirus

**DOI:** 10.3390/v11050421

**Published:** 2019-05-06

**Authors:** Min Feng, Feifei Ren, Yaohong Zhou, Nan Zhang, Qiuyuan Lu, Luc Swevers, Jingchen Sun

**Affiliations:** 1 Guangdong Provincial Key Laboratory of Agro-animal Genomics and Molecular Breeding, College of Animal Science, South China Agricultural University, Guangzhou 510642, China; hunanfengmin@163.com (M.F.); zzff43@outlook.com (F.R.); 18825075438@163.com (Y.Z.); wan850793534@126.com (N.Z.); 18819427778@163.com (Q.L.); 2 Insect Molecular Genetics and Biotechnology, Institute of Biosciences and Applications, National Centre for Scientific Research Demokritos, Aghia Paraskevi, Athens 15341, Greece

**Keywords:** *Antheraea pernyi*, LTR-retrotransposons, ApNPV

## Abstract

The published genome sequence of *Antheraea* *yamamai* (Saturnnidae) was used to construct a library of long terminal repeat (LTR)-retrotransposons that is representative of the wild silkmoth (*Antherea)* genus, and that includes 22,666 solo LTRs and 541 full-length LTRs. The LTR retrotransposons of *Antheraea* *yamamai* (AyLTRs) could be classified into the three canonical groups of *Gypsy*, *Copia* and *Belpao*. Eleven AyLTRs contained the *env* gene element, but the relationship with the *env* element of baculovirus, particularly *A*. *yamamai* and *pernyi* nucleopolyhedrovirus (AyNPV and ApNPV), was distant. A total of 251 “independent” full-length AyLTRs were identified that were located within 100 kb distance (downstream or upstream) of 406 neighboring genes in *A*. *yamamai*. Regulation of these genes might occur in *cis* by the AyLTRs, and the neighboring genes were found to be enriched in GO terms such as “response to stimulus”, and KEGG terms such as “mTOR signaling pathway” among others. Furthermore, the library of LTR-retrotransposons and the *A*. *yamamai* genome were used to identify and analyze the expression of LTR-retrotransposons and genes in ApNPV-infected and non-infected *A*. *pernyi* larval midguts, using raw data of a published transcriptome study. Our analysis demonstrates that 93 full-length LTR-retrotransposons are transcribed in the midgut of *A*. *pernyi* of which 12 significantly change their expression after ApNPV infection (differentially expressed LTR-retrotransposons or DELs). In addition, the expression of differentially expressed genes (DEGs) and neighboring DELs on the chromosome following ApNPV infection suggests the possibility of regulation of expression of DEGs by DELs through a *cis* mechanism, which will require experimental verification. When examined in more detail, it was found that genes involved in Notch signaling and stress granule (SG) formation were significantly up-regulated in ApNPV-infected *A*. *pernyi* larval midgut. Moreover, several DEGs in the Notch and SG pathways were found to be located in the neighborhood of particular DELs, indicating the possibility of DEG-DEL cross-regulation in *cis* for these two pathways.

## 1. Introduction

Sericulture is one of the great inventions of the ancient Chinese, and it has created enormous economic benefits for society by the art of silk production. Chinese oak silkworm, *Antheraea pernyi* (Lepidoptera: Saturniidae), is an important agricultural wild silkworm species and also a source of food with high-quality protein. It was reported that the protein content of silkworm pupae represents all of the amino acids needed by the human body [1]. Indeed, *A*. *pernyi* at various life stages such as fifth-instar larvae, adults and pupae can be served as food for human consumption by sauteing, frying, boiling, or roasting [2]. Today, as increasing attention is paid to healthy diets, silkworm as a source of food will receive more and more interest. However, *A*. *pernyi* larvae are raised on oak leaves in the field. Thus, their growth and development are threatened by numerous pathogens including viruses, bacteria, microsporidia and fungi [3,4,5,6].

Among the pathogenic microorganisms that harm *A*. *pernyi*, the loss caused by baculovirus (nucleopolyhedrosis virus or NPV) is enormous. *A*. *pernyi* NPV (ApNPV) belongs to the group I of lepidopteran NPVs, which displays two types of phenotypes during the infection cycle, namely occlusion-derived virus (ODV) and budded virus (BV) [7,8]. The average incidence rate of nuclear polyhedrosis disease induced by ApNPV averages approximately 30% and occasionally reaches above 70% [7]. *A*. *pernyi* larvae infected with ApNPV exhibit characteristic symptoms at the late stage of infection including cessation of feeding, spots of damage on the epidermis and abnormal behavior, leading to death [3]. Indeed, ApNPV infections have brought huge economic losses to sericulture in various countries. However, the mechanisms underlying the interaction between ApNPV and the host *A*. *pernyi* are still not clear.

The long terminal repeat (LTR) retrotransposons are among the most abundant constituents of eukaryotic genomes [9]. Full-length LTR-retrotransposons consist of direct sequence repeats of varying structure at their ends that encompass different (from one to three) open-reading frames (ORFs) such as *gag*, *pol* and *env* [10]. In addition, numerous solitary LTRs, that result from the loss of ORFs between the two flanking LTRs, can exist in the host genome. Generally, LTR-retrotransposons are thought to be silent. However, many LTR-retrotransposons, especially the elements of endogenous retrovirus, were found to be transcriptionally active [11,12]. Furthermore, many studies have found that the elements of LTR-retrotransposons can be involved in disease development and the host immune response [12,13,14]. However, to our knowledge, no reports exist on a role for LTR-retrotransposons acting as host factors to affect the process of viral infection in insects.

Given that the genome of *A*. *pernyi* is not available, the genome of the closely related Japanese oak silk moth (*A*. *yamamai*) was used for the analysis of LTR-retrotransposons [15]. *A*. *yamamai* also belongs to the Saturniidae family and is a sibling species of *A*. *pernyi* [16]. Thus, using the *A*. *yamamai* genome as a reference genome [15] and the transcriptome raw data of the midgut *of A*. *pernyi* in the absence or presence of ApNPV infection [3], we provide an overview of the expression of LTR-retrotransposons and their neighbouring genes in the midgut of ApNPV-infected *A*. *pernyi* larvae.

## 2. Materials and Methods

### 2.1. Identification and Characterization of LTR-Retrotransposons

To identify LTR-retrotransposons, the *A*. *yamamai* genome was used as a reference genome and downloaded from the NCBI database (project accession PRJNA383008 and PRJNA383025) [15]. LTR-retrotransposons were identified in the *A*. *yamamai* genome using the software LTRharvest (GenomeTools1.5.7) and LTRdigest [17,18]. Pairs of putative LTRs that were separated by 1–15 kb and flanked by target site duplications were screened by LTRharvest in *A*. *yamamai* genome. The threshold of LTR nucleotide similarity used in LTRharvest was set at higher than 80%; other parameters were set to their defaults. Internal features of LTR-retrotransposons, including protein domains, primer-binding sites, and polypurine tracts, were predicted using LTRdigest with default setting.

In the present study, LTR-retrotransposons which contain at least one relevant protein domain between the pairs of putative LTRs are called full-length LTR-retrotransposons. Correspondingly, LTR-retrotransposons lacking all protein domains are called solo LTR-retrotransposons.

### 2.2. Classification and Phylogenetic Analysis of LTR-Retrotransposons

The superfamily classification of LTR-retrotransposons of *A*. *yamamai* (AyLTR) is based on the homology between their reverse transcriptase (RT) domain sequences and the sequences of Peptidase_A17, RVT_1 and RVT_2 from the Pfam database [19]. Sequences of the RT domain from LTR-retrotransposons were used for multiple alignment using MUSCLE (v3.8.31) [20]. A phylogenetic tree built based on the neighbor-joining algorithm was generated from the RT domain alignment using MEGA5 with 1000 bootstrap replicates.

In addition, according to the positional relationship between full-length LTR-retrotransposons and host genes, LTR-retrotransposons are divided into three categories in our study. Full-length LTR-retrotransposons that do not overlap with gene exon sequences are defined as independent LTRs (“Stream”); full-length LTR-retrotransposons that partially overlap with a gene exon sequence are defined as partially overlapping LTRs (“Part”); and full-length LTR-retrotransposons that encompass gene exon sequences are defined as overlapping LTRs (“In”).

### 2.3. Analysis of the Env Genes from AyLTRs

AyLTRs possessing *env*-like elements were selected from the library of full-length LTR-retrotransposons. The fusogenic region of AyLTR envelope glycoproteins was analyzed using MegAlign (DNASTAR Lasergene.v7.1) and Weblogo (http://weblogo.berkeley.edu/logo.cgi). Phylogenetic analysis of LTR-retrotransposon *env* sequences from different insect species was performed using MegAlign and MEGA5 (5.0.1.102). In addition, sequence alignments were performed between AyLTR *env* genes and genes that code for envelope proteins (F and GP64) in Group I and Group II nuclear polyhedrosis viruses (NPVs) using MegAlign and MEGA5 with 1000 bootstrap replicates.

Following LTR-retrotransposons and NPV strains were analyzed: (1) Insect LTR-retrotransposons (endogenous retroviruses or ERVs): *Drosophila melanogaster* tirant virus (tirant, X93507), *Drosophila melanogaster* ZAM virus (ZAM, AJ000387), *Trichoplusia ni* TED virus (TED, M32662), *Drosophila melanogaster* nomad virus (nomad, AF039416), *Drosophila melanogaster* Gypsy virus (DmeGypV, M12927) and *Drosophila melanogaster* Idefix virus (Idefix, AJ009736); (2) BmERVs, which were identified in our previous study [21] ; (3) Group I NPVs: *Bombyx mori* nucleo polyhedrosis virus (BmNPV) (strains JapanH4: LC150780.1, Suzhou Cubic: JQ991009.1), *Autographa californica* nucleo polyhedrosis virus (AcMNPV) cloneC6 (L22858.1), AcMNPV E2 (KM667940.1), *Orgyia pseudotsugata* multinucleocapsid nucleo polyhedro virus (OPMNPV, U75930.2), *Antheraea yamamai* nucleopolyhedrovirus (AyNPV, LC375537.1) and *Antheraea pernyi* nucleopolyhedrovirus (ApNPV, strain liaoling, LC194889.1); (4) Group II NPVs: *Helicoverpa armigera* single nucleo polyhedro virus (HaSNPV) (NC_003094.2), *Lymantria dispar* multiple nucleopolyhedrovirus (LdMNPV)-RR01(KX618634.1), LdMNPV-Ab-a624 (KT626572.1), *Spodoptera exigua* multiple nucleopolyhedrovirus (SeMNPV) HT-SeG25(HG425347.2) and SeMNPV VT-SEOX4 (HG425345.1).

### 2.4. Identification of Genes Located in the Neighborhood of AyLTRs in the A. yamamai Genome

The UCSC Genome Bioinformatics tool was used to screen for genes located within 100 kb of upstream and downstream of full-length AyLTR elements. Target genes are defined as genes that have annotated exons (UTR and CDS) within the defined sequence space of 100 kb.

Blast2GO and WEGO were used to perform gene ontology (GO) classification. The GO terms included molecular function, cellular component and biological process. For the pathway enrichment analysis, the genes were mapped to Kyoto Encyclopedia of Genes and Genomes (KEGG) database. The hypergeometric test was used to identify overrepresented KEGG pathway and GO terms with a significance level of *p* < 0.05.

### 2.5. Analysis of RNA-Seq Data

To analyze the LTR-retrotransposon transcriptome in *A*. *pernyi* infected with ApNPV, we downloaded the raw reads from NCBI with the accession numbers: SRR2919240, SRR2919241, SRR2919242 and SRR2919243 [3]. In the study that provided the transcriptome raw data for our analysis [3], *A*. *pernyi* larvae infected with 4.05 × 10^6^ polyhedra/mL ApNPV for three days were the experimental group while the control group was treated with the same volume of 0.9% NaCl physiological saline. Each group included two independent biological replicates. The four cDNA libraries were designated as Ap_CK1, Ap_CK2, Ap_NPV1 and Ap_NPV2 [3].

These raw reads were mapped to the *A*. *yamamai* genome using TopHat2 (version 2.0.3.12) [22]. The mapped reads of each sample were assembled by software Cufflinks and Cuffmerge. Gene and LTR-retrotransposon abundances were quantified by software RSEM [23] and their expression level was normalized by FPKM (Fragments Per Kilobase of transcript per Million mapped reads).

To identify differentially expressed genes (DEGs) and differentially expressed LTR-retrotransposons (DELs), the edgeR package (http://www.rproject.org/) was used. Genes and LTR-retrotransposons with fold change of |log_2_^FC^| > 1 (FC: fold change) and a false discovery rate (FDR) <0.05 were considered as DEGs and DELs. The interaction networks of DELs and neighboring DEGs were imported to Cytoscape software for visualization.

## 3. Results

### 3.1. De novo Detection of AyLTRs in the Antheraea yamamai Genome

AyLTRs were detected by LTRharvest and annotated with LTRdigest. A total of 23,207 AyLTRs were identified in the *A*. *yamamai* genome. Among these are 541 full-length AyLTRs and 22,666 solo AyLTRs. Furthermore, we identified 11 AyLTRs containing the *env* ORF. The sizes of the 541 full-length AyLTRs varied from 1127 to 15,130 bp and the lengths of the LTR ranged from 100 to 1000 bp. Details of the AyLTRs are shown in Supplementary Materials Table S1.

### 3.2. Phylogenetic Analysis and Classification of Full-Length AyLTRs

**Figure 1:**
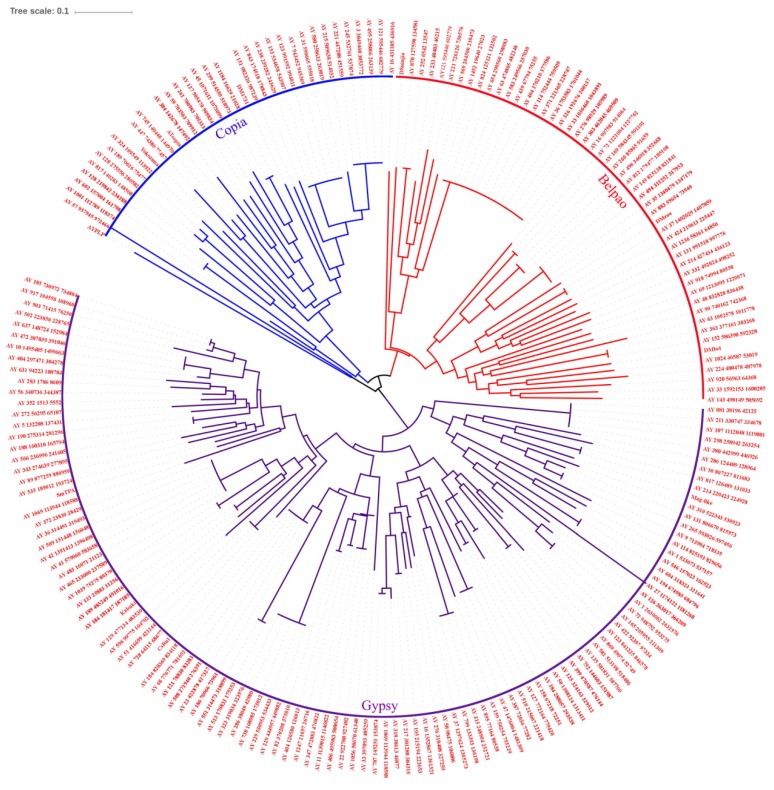
Phylogenetic tree of full-length AyLTRs based on the alignment of the RT region. A total of 189 AyLTRs contained an RT domain with sufficient conservation for confident alignment prior to phylogenetic analysis. Other reference LTRs belonging to *Copia*, *Belpao* and *Gypsy* that were used in the analysis, include: AYPLP (AY217340), Yokozuna (AB014676), Atcopia (BAB09923), DM1731 (X07656), Dsninjia (AB042129), DMroo (AY180917), Dmbel (U23420), Mag-like (AB126055), CsRn1(AY013563), Kabuki (AB032718) and SmTPA (DAA04495). Topology was based on the neighbor-joining method with 1000 bootstraps. In the phylogenetic tree, the *Copia* (blue), *Belpao* (red), and *Gypsy* (purple) clades can be distinguished.

To classify the full-length AyLTRs, their RT domain sequences and those of other insect LTRs were used to build a multiple alignment and compute phylogenetic trees using the neighbor-joining method of MEGA 5. Among the 541 full-length AyLTRs, 189 AyLTRs had an RT domain that was sufficiently conserved for confident alignment during phylogenetic analysis. AyLTRs were classified into the three canonical groups *Gypsy*, *Copia* and *Belpao* (Figure 1). Most of the AyLTRs included in the phylogenetic analysis belonged to the *Gypsy* group (Figure 1).

### 3.3. Analysis of Env Genes from Full-Length AyLTRs

**Figure 2:**
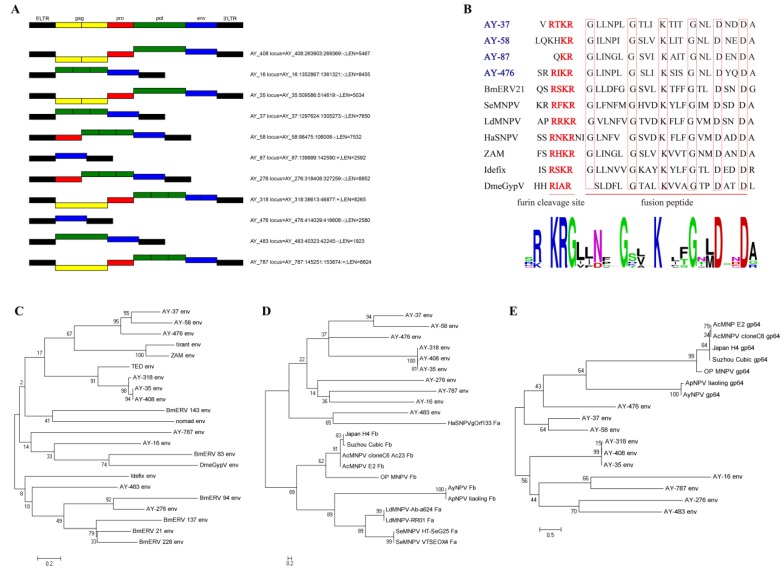
Analysis of the AyLTRs *env* genes. (**A**) Structure of AyLTRs which contained the *env* element. (**B**) Multiple alignment of the conserved amino acid sequence block shows a consensus furin cleavage site RxxR and the peptide fusion consensus sequence in the envelope proteins of AY-37, AY-58, AY-87, AY-476, BmERV21, ZAM, Idefix, DmeGypV, and the F protein of SeMNPV, LdMNPV and HaSNPV. Logo visualization of the furin cleavage site and peptide fusion consensus sequence was performed using WEBLOGO (weblogo.berkeley.edu/logo.cgi). (**C**) Phylogenetic analysis of the *env* gene of AyLTRs and known insect LTR retrotransposons. (**D**) Phylogenetic analysis of the *env* gene of AyLTRs and F genes from Group I NPVs (Fb) and Group II NPVs (Fa). (**E**) Phylogenetic analysis of the *env* gene of AyLTRs and GP64 of Group I NPVs.

Eleven full-length AyLTRs containing the *env* gene element were identified in the *A*. *yamamai* genome (Figure 2A). Only four AyLTRs, namely AY-408, AY-35, AY-318 and AY-787, contained the complete structure (LTR-gag-pro-pol-env-LTR) of an insect retrovirus (Figure 2A). We further found that the *env* amino acid sequences of AY-37, AY-58, AY-87 and AY-476 shared a region of similarity with the fusion proteins of known insect ERVs and Group II NPVs, such as the furin cleavage signal and the downstream fusion peptide (Figure 2B). Logo representation of the furin-like consensus motif is RxxR and the peptide fusion consensus sequence is GxxxxxGxxxKxxxGxxDxxD (Figure 2B). However, the furin cleavage site located in front of the fusion peptide in AY-58 and AY-87 is incomplete (Figure 2B). On the other hand, the results suggest that the *env* genes of AY-37 and AY-476 have the potential to encode fusion proteins.

Phylogenetic analysis shows that the nucleotide sequences of *env* of AY-318, AY-35 and AY-408 are closely related with *env* of *Trichoplusia ni* TED virus. The AY-276 *env* was closely related to BmERV 94 *env*. In addition, close phylogenetic relationships of *env* genes were also observed between AY-37, AY-58, AY-476 and *Drosophila melanogaster* tirant and ZAM ERVs (Figure 2C).

To explore the evolutionary relationship between AyLTR *env* genes and genes encoding the envelope protein (Env) in Group I and II NPVs, corresponding phylogenetic trees were generated. The results showed that the *env* sequences of AyLTRs were distantly related to *Fa*, *Fb* and *gp64* genes from AyNPV, ApNPV, as well as other Group I and Group II NPVs (Figure 2D, E). On the other hand, AY-483 *env* was clustered on the same branch of the phylogenetic tree with HaSNPVgOrf133 *Fa* (Figure 2D).

### 3.4. Identification and Analysis of (Potentially Cis-Target) Genes that Occur in the Neighborhood of AyLTRs

**Figure 3:**
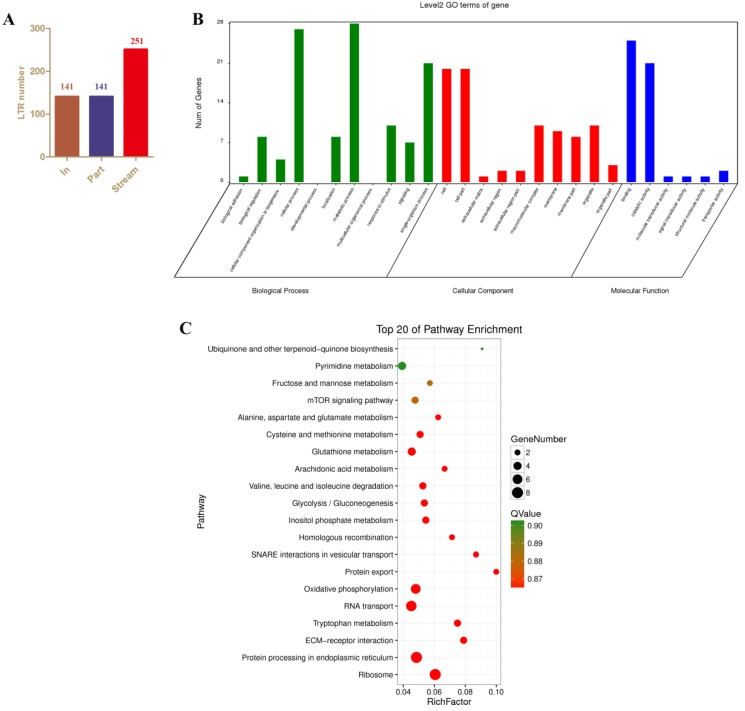
GO and KEGG analysis of the neighboring genes of independent full-length AyLTRs (“Stream”). (**A**) According to the positional relationship between AyLTRs and their neighboring genes, AyLTRs were classified into three types, “In”, “Part” and “Stream”. “In” represent AyLTRs that encompass gene exon sequences. “Part” represents AyLTRs that partially overlap with exon sequences. “Stream” represents independent AyLTRs that do not overlap with host gene exons. (**B**) Histogram description of Gene Ontology enrichment of neighboring genes of “Stream” AyLTRs. Genes were assigned to three categories: biological process (BP), cellular component (CC), and molecular function (MF). (**C**) The neighboring genes of “Stream” AyLTRs that were enriched in KEGG pathways. Genes located ∼100 kb upstream and downstream of “Stream” AyLTR were identified as “neighboring” genes.

To identify host genes that may be regulated by LTR-retrotransposons, we analyzed the positional relationship between AyLTRs and their neighboring genes. A total of 141 “In” AyLTRs were identified that encompassed exons of host genes and 141 “Part” AyLTRs were detected that overlapped with neighboring exons (Figure 3A). Among these AyLTRs, six AyLTRs belong to both “In” and “Part” categories. In addition, 251 full-length “Stream” AyLTRs were identified as independent LTRs (Figure 3A). 14 AyLTRs are located on scaffolds that do not contain genes.

“Stream” AyLTRs were further examined whether their expression was coordinated with neighboring cellular genes which could be indicative for regulation of expression of cellular genes in *cis* by the LTRs [24,25]. Applying a method for finding *cis*-target genes of lncRNAs [26], 406 genes located within 100 kb upstream and downstream of “Stream” AyLTRs were identified as potential *cis*-target genes of these AyLTRs. GO biological process analysis showed that most of the candidate *cis*-target genes were enriched in “cellular process”, “metabolic process”, “response to stimulus” and “single-organism process” (Figure 3B). KEGG analysis illustrated mainly enrichment in “oxidative phosphorylation”, “RNA transport”, “Protein processing in endoplasmic reticulum”, “mTOR signaling pathway” and several metabolic pathways (Figure 3C); see Supplementary Materials Table S2 for more details).

### 3.5. Transcriptome Analysis of LTR-Retrotransposons in A. pernyi Larval Midgut Samples

To obtain a global view of LTR-retrotransposon expression of the *A*. *pernyi* larval midgut in response to ApNPV infection, the *A*. *yamamai* genome was first used as a reference genome to extract LTR-retrotransposon sequences from the raw transcriptome data of the study described in [3]. After discarding adaptor and low-quality reads, 71,599,652, 57,723,648, 70,342,118 and 61,942,502 clean reads were obtained from four cDNA libraries of *A*. *pernyi* midgut (two controls and two ApNPV-infected samples; SRR2919240, SRR2919241, SRR2919242 and SRR2919243) (Table 1) [3]. The clean reads were mapped onto the *A*. *yamamai* reference genome, and the mapping rate of each library ranged from 63.31–67.39% (Table 1).

**Figure 4:**
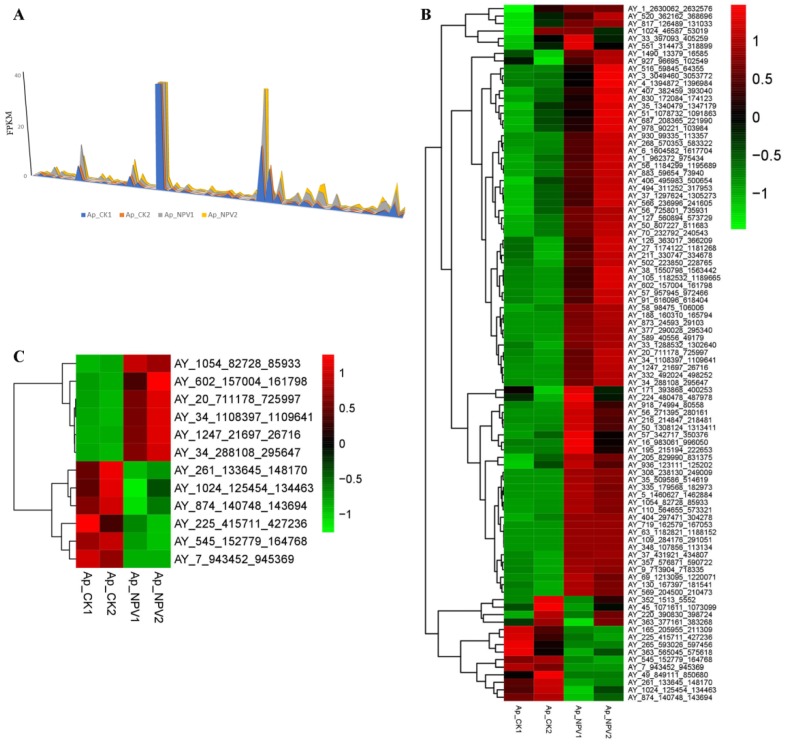
Expression of LTR-retrotransposons in ApNPV-infected *A*. *pernyi* larval midgut and uninfected control samples. (**A**) RNA sequence data from ApNPV-infected *A*. *pernyi* larval midgut and uninfected controls [3] were aligned using TopHat and transcripts were constructed using Cufflinks. The number of fragments per kilobase per million reads (FPKM) was plotted against the AyLTRs. (**B**) Heatmap of expression of LTR-retrotransposons in ApNPV-infected *A*. *pernyi* larval midgut samples and their uninfected controls after analysis of RNA sequence data. (**C**) Heatmap of differentially expressed LTR-retrotransposons (DELs) in ApNPV-infected *A*. *pernyi* larval midgut samples.

The analysis resulted in the identification of 93 full-length LTR-retrotransposons that were transcribed in ApNPV-infected *A*. *pernyi* larval midgut samples or their controls (Figure 4A). The expression of these LTR-retrotransposons in the four *A*. *pernyi* midgut samples are presented in the heatmap of Figure 4B. Compared to uninfected controls, most of LTR-retrotransposons were up-regulated in ApNPV-infected samples (Figure 4B). However, a considerable number of LTR-retrotransposons exhibit variable expression between the two samples in each category, especially in the samples after infection with ApNPV. LTRs that expressed inconsistencies in expression between samples in each category were removed, and finally only the LTR-retrotransposons that are consistently down- or up-regulated in each sample were used for subsequent analysis. Details of the expression of these LTR-retrotransposons are presented in Supplementary Materials Table S3.

**Figure 5:**
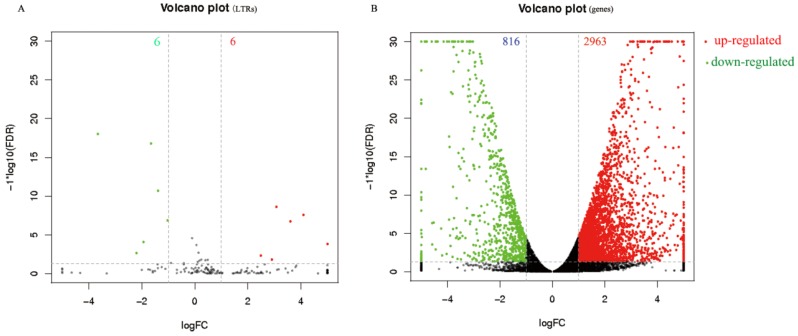
Volcano plot of identified DELs and DEGs between ApNPV-infected *A*. *pernyi* larval midgut and uninfected controls. (**A**) Volcano plot of differentially expressed LTRs (DELs). (**B**) Volcano plot of differentially expressed genes (DEGs). The red spots represent significantly up-regulated DELs and DEGs. The green spots represent significantly down-regulated DELs and DEGs. The black spots indicate absence of significantly different expression.

Furthermore, on the basis of the differential expression analysis, 12 significant DELs were identified between the ApNPV-infected and uninfected midgut samples, of which six were up-regulated and six were down-regulated (Figure 4C, Figure 5A; Table 2). In addition, compared to the uninfected control, 2963 up-regulated DEGs and 816 down-regulated DEGs were identified in ApNPV-infected *A*. *pernyi* larval midgut samples (Figure 5B).

**Figure 6:**
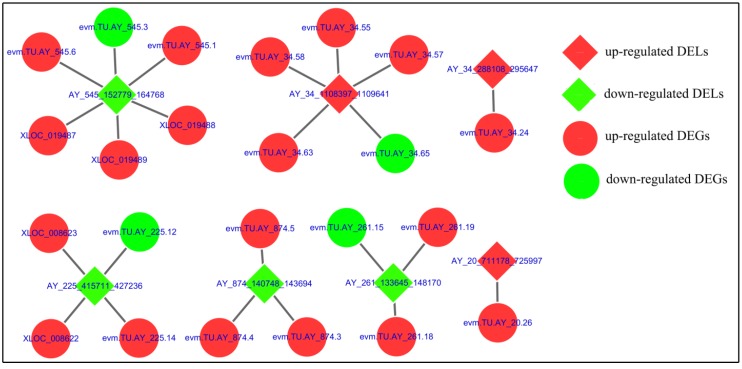
DELs-DEGs network obtained after comparison of ApNPV-infected *A*. *pernyi* samples with uninfected controls. DELs and their corresponding neighboring (putative *cis*-target) DEGs were used to construct a DELs-DEGs interaction network. In this network, up-regulated and down-regulated genes are displayed as red and green circles, respectively; similarly, up-regulated and down-regulated LTRs are displayed as red and green squares.

To further explore the connection between DELs and DEGs, we examined whether adjacent putative *cis*-targets of DELs could correspond to DEGs during ApNPV infection. On the basis of this analysis, a DELs-DEGs interaction network was constructed using Cytoscape (Figure 6). In the network, 9 DELs-DEGs connections were positively correlated, whereas another 14 connections were negatively correlated (Figure 6). The specific description of these 23 DEGs is shown in Table 3.

### 3.6. Bioinformatics Analysis of Notch Signaling and Stress Granule (SG) Regulation Pathways

**Figure 7:**
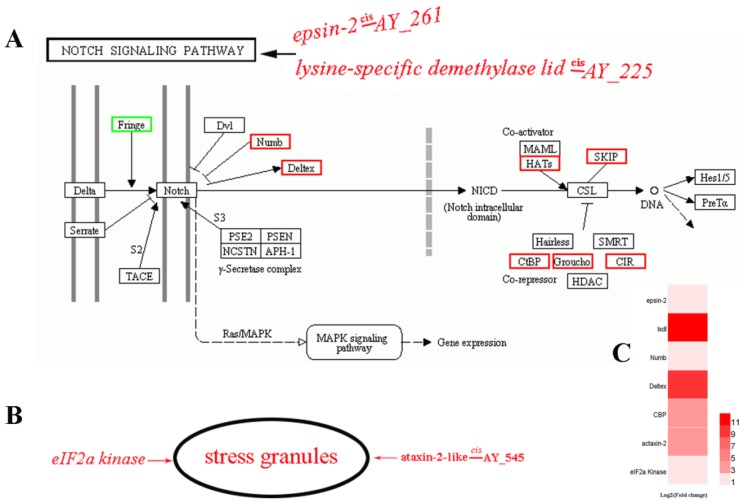
DEGs that are located in the neighborhood (putative *cis*-targets) of DELs and that are associated with Notch signaling pathway and stress granule formation. (**A**) Some DEGs including Numb, Deltex, CBP, Epsin-2 and lysine-specific demethylase lid (*lsdl*) that are associated with Notch signaling are increased in expression during ApNPV infection. As also shown in Figure 6, epsin-2 (evm.TU.AY_261.18) and lysine-specific demethylase lid (XLOC_008622) are considered as putative *cis*-targets of AY_261_133645_148170 and AY_225_415711_427236, respectively, in *A*. *pernyi*. Therefore, we speculate that AY-261 and AY-225 might participate in the regulation of the Notch signaling pathway by regulating the expression of epsin-2 and lysine-specific demethylase lid by a mechanism in *cis*. (**B**) Stress granule formation can occur as a result of eIF2α phosphorylation caused by eIF2a kinase. eIF2a kinase expression was activated during ApNPV infection in *A*. *pernyi*. Ataxin-2-like protein, a neighboring gene and potential *cis*-target of AY_545, is a component of stress granules and could possibly function as a regulator of stress granules during ApNPV infection. (**C**) Heat map of the relative expression of epsin-2, lsdl, Numb, Deltex, CBP, ataxin-2-like protein and eIF2a kinase in ApNPV-infected *A*. *pernyi* compared with uninfected controls.

During further analysis, attention was focused on up-regulated DEGs involved in Notch signaling and stress granule (SG) regulation (Figure 7) (see also discussion). These up-regulated DEGs included deltex, CREB-binding protein (*CBP*), protein numb (NUMB), epsin-2, lysine-specific demethylase lid (lsdl), ataxin-2-like protein and eIF2a kinase (Figure 7C).

Interestingly, DEGs involved in Notch signaling pathway including epsin-2 and lysine-specific demethylase lid (lsdl) were adjacent to AY_261_133645_148170 and AY_225_415711_427236, respectively, and might be *cis*-regulated by these DELs (Figure 6 and Figure 7). Additionally, ataxin-2, an SG-related DEG, was also identified as a putative *cis*-target of AY_545_152779_164768 (Figure 6 and Figure 7).

## 4. Discussion

In the present study, we attempted to explore the role of LTR-retrotransposons in the process of ApNPV infection in the wild silkmoth *A*. *pernyi*. However, genome-wide detection of LTR-retrotransposons in *A*. *pernyi* has not been performed due to the deficiency of genomic data. Fortunately, the annotated genome sequence of *A*. *yamamai*, the first published genome within the *Saturniidae* family, was released recently [15]. *A*. *pernyi* and *A*. *yamamai* are sibling species within the *Antheraea* genus and the *Saturniidae* family [27]. Given that no *A*. *pernyi* genome is currently available, the genome of *A*. *yamamai* is used as reference genome in this study. Indeed, the clean reads of *A*. *pernyi* transcriptome could be mapped confidently onto the *A*. *yamamai* reference genome. The mapping rate of each library ranged from 63.31–67.39%.

For our analysis, a library of LTR-retrotransposons of wild silkworm (*Antherea*) was first constructed after screening of the *A*. *yamamai* genome with LTRharvest and LTRdigest. The obtained library contained 541 full-length AyLTRs and 22,666 solo AyLTRs. Based on a comparative analysis of the conserved RT domain, AyLTRs could be grouped into the canonical phylogenetic clades of *Gypsy*, *Copia*, and *Belpao* [28].

However, among 541 full-length AyLTRs, only eleven AyLTRs contained the *env* element. The infectious ability of insect LTR-retrotransposons is thought to be associated with the expression of the envelope protein encoded by the *env* gene [29]. Thus far, only the well-known *gypsy* and *ZAM* elements of *D*. *melanogaster* have been shown to possess infectious properties [30,31]. Furthermore, it was reported that the *env* gene of LTR-retrotransposons was evolutionary related with the envelope fusion protein lineage of baculovirus [32,33]. The region of the envelope fusion protein with the highest sequence similarity with insect LTR-retrotransposons includes the furin hydrolysis signal and a fusion peptide located downstream [33]. In our study, we observed that the envelope fusion protein of AY-476 and AY-37 possessed a complete furin cleavage site with a downstream fusion peptide, whereas in AY-58 and AY87 only a partial furin cleavage site was detected. These results suggest that particular AyLTRs with *env* gene elements, such as AY-476 and AY-37, might possess infectious properties. It is believed that some LTR-retrotransposons which lacked the *env* gene, became integrated into the dsDNA genome of a baculovirus from which they could “capture” the *env* gene [32]. However, we found that the *env* sequences of AyLTRs only had a distant relationship with genes encoding F (Fa and Fb) and GP64 protein from Group I and Group II NPVs in general, and more specifically from ApNPV and AyNPV. These results indicate that the *env* elements were not derived from the envelope gene of ApNPV and AyNPV.

It is well documented that LTR-retrotransposons can affect the physiopathology of host cells at multiple levels. For instance, LTR-retrotransposons can modulate the expression of adjacent host genes in the human genome [34]. While the expression of LTR-retrotransposon proteins with conventional retroviral functions can influence the host’s physiological or pathological states [35], it was also reported that non-coding LTR-retrotransposons can be biologically active [36]. To investigate interactions between LTR-transposons and cellular genes in *cis*, we first identified the host genes that are adjacent to AyLTRs within the *A*. *yamamai* genome. It was assumed that some of these AyLTRs may be involved in host biological responses through regulation of the expression of host genes.

Based on the information on AyLTRs and their neighboring genes, it was attempted to decipher the biological function of AyLTR during ApNPV infection of *A*. *pernyi* by expression analysis. Due to the absence of genomic data of *A*. *pernyi*, the published transcriptome analysis of the *A*. *pernyi* infected with ApNPV was *de novo* assembled using the Trinity platform in the published study [3]. In this study, on the other hand, the transcriptome of LTR-retrotransposons and genes was analyzed after mapping of the reads to the *A*. *yamamai* genome. Using this approach, 93 full-length LTR-retrotransposons were found to be transcribed in ApNPV-infected *A*. *pernyi* larval midgut samples or their uninfected controls. Interestingly, six LTR-retrotransposons were significantly up-regulated, and six LTR-retrotransposons were significantly down-regulated in ApNPV-infected *A*. *pernyi* midgut. Whether these 12 DELs can play a functional role during ApNPV infection of *A*. *pernyi* will require further experimentation. Moreover, interactions between 7 DELs and their 23 adjacent host DEGs were identified during ApNPV infection. We speculate that the expression of the 23 identified DEGs is under the regulatory control of the seven DELs by a mechanism of interaction in *cis* along the chromosome during ApNPV infection in *A*. *pernyi*. Indeed, an involvement in host-virus interaction as well as immune response processes is very well documented in the case of endogenous retroviruses, a special category of LTR-retrotransposons [37,38,39].

For a few of these 23 DEGs, a role in the response of the virus-infected host was observed in the silkworm *Bombyx mori*. The expression of reverse transcriptase (RT) was up-regulated during BmNPV infection in BmNPV resistant silkworm strains [40]. The up-regulation of *RT* in *A*. *pernyi* midgut during ApNPV infection confirms its possible role in host antiviral response. Conversely, the expression of *Tret1* was down-regulated in *A*. *pernyi* midgut during ApNPV infection, which fits with previous research documenting that down-regulation of trehalose transporter Tret1-like might enhance infection of BmNPV in *B*. *mori* [40].

For a more in-depth analysis of a subset of our data, attention was focused on a possible role of the Notch signaling pathway and stress granule regulation during ApNPV infection of the midgut of *A*. *pernyi* and the possible involvement of their regulation by LTR-retrotransposons.

The Notch signaling pathway is important in development, tissue homeostasis, as well as disease [41], including viral disease [42,43]. In this study, several up-regulated DEGs were found to be associated with Notch signaling during ApNPV infection (Figure 7A). Among these DEGs, the homolog of *deltex 1* is involved in T cell immunity in mammals [44]. *CREB-binding protein* (*CBP*) encodes a coactivator protein (histone acetyltransferase or HAT) that plays a role in the innate antiviral immunity pathway in vertebrates [45] and is also targeted by virus-encoded suppressor mechanisms [46]. In the LNX/Numb/Notch pathway, Numb protein (DEG during ApNPV infection) was also identified as a target of the Np9 protein encoded by the human endogenous retrovirus K [47]. Thus, we speculate that Notch signaling-related DEGs such as deltex, CBP and Numb might be associated with host responses in *A*. *pernyi* against ApNPV.

In addition, epsin-2 and lysine-specific demethylase lid were up-regulated after ApNPV infection in our analysis (Table 3). Coincidentally, these two genes were found to play an important role in Notch signaling [48,49]. Moreover, epsin-2 and lysine-specific demethylase lid are putative *cis*-targets of AY_261_133645_148170 and AY_225_415711_427236, respectively in *A*. *pernyi*. Based on these interesting connections, we forward the hypothesis that expression of important genes in Notch signaling in the host can be regulated by LTR-retrotransposons, which in turn are responsive to ApNPV infection.

In mammalian systems, ataxin-2-like protein encodes a component of stress granules (SG) that also regulates *p*-body (PB) formation [50]. SGs and PBs provide cell homeostasis and mRNA stability during the stress response induced by viral infection [51]. More importantly, SGs play a critical role in the host antiviral immune response. SG formation can interfere with viral replication because all viruses require the host translation machinery to synthesize viral proteins. Accordingly, many viruses have evolved diverse mechanisms to inhibit SG formation. Antiviral functions of SGs include the establishment of an antiviral state by limiting viral protein accumulation and the regulation of signaling cascades that affect virus replication and immune responses [52]. In mammals, SG formation can occur as a result of eIF2α phosphorylation caused by diverse eIF2a kinases activated by different stress conditions [51]. Interestingly, the expression of *eIF2a kinase* was also up-regulated in *A*. *pernyi* after ApNPV infection (Figure 7C). Accordingly, we speculate that differential expression of ataxin-2-like protein and eIF2a kinase reflects the regulation of SG formation during ApNPV infection in *A*. *pernyi* (Figure 7B). Moreover, given that ataxin-2 has been identified as a putative *cis*-target of AY_545_152779_164768 (Table 3), the possibility is considered that AY_545 is involved in the regulation of stress granule formation during ApNPV infection.

**Figure 8:**
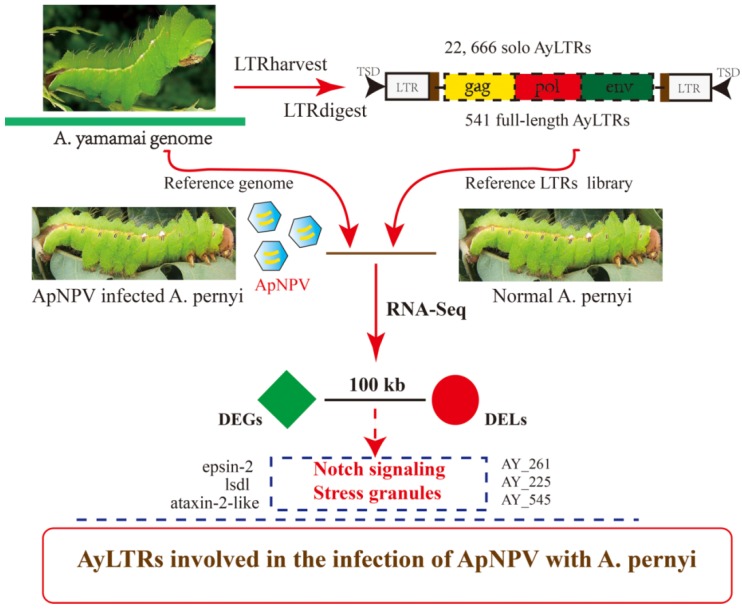
General overview of the study and its conclusions. Based on the genome of *Antheraea yamamai* [15], which is the first draft genome in the family of saturniid moths, a wild silkworm LTR-retrotransposons library was constructed of 22,666 solo LTRs and 541 full-length LTRs. Using this information, published RNA sequence raw data of ApNPV-infected *A*. *pernyi* larval midgut and uninfected control samples [3] were analyzed to identify 12 DELs and 3779 DEGs with respect to ApNPV infection. In more detailed analysis, several DEGs, that are associated with Notch signaling and stress granule formation, are considered to be regulated in *cis* by DELs during ApNPV infection (Dotted line). Our study indicates a potential role for LTR-retrotransposons to regulate the host gene response during ApNPV infection of *A*. *pernyi*.

As summarized in Figure 8, a wild silkworm LTR-retrotransposons library was established based on the first draft genome in the family of Saturniidae, which included 22,666 solo LTRs and 541 full-length LTRs. Using the *A*. *yamamai* genome as a reference genome [15] and published raw data of the transcriptome of ApNPV-infected *A*. *pernyi* midgut [3], 93 full-length LTR-retrotransposons were found to be transcribed in ApNPV-infected *A*. *pernyi* larval midgut samples or their uninfected controls. Candidate DEGs were identified, including epsin-2, lysine-specific demethylase lid and ataxin-2-like protein that are involved in Notch signaling and stress granule formation, that could be subject to regulation in *cis* by DELs in *A*. *pernyi* during ApNPV infection. Further experimentation is required to verify whether LTR-retrotransposons are involved in the response of *A*. *pernyi* to ApNPV by regulating particular genes associated with Notch signaling and stress granule formation. The functional relevance of other DEL-DEG combinations identified by bioinformatics likewise needs experimental validation in future studies.

## Figures and Tables

**Table 1:** RNA-Seq data statistics.

Sample Name	Raw Reads	Clean Reads	Total Mapped	All Gene Num
Ap_CK1	74,427,158	71,599,652	66.90%	10,535
Ap_CK2	59,939,546	57,723,648	67.39%	10,334
Ap_NPV1	73,741,696	70,342,118	64.60%	11,041
Ap_NPV2	64,399,928	61,942,502	63.31%	11,152

**Table 2:** DELs that are differentially expressed during ApNPV infection.

ID	Ap_CK_fpkm	Ap_NPV_fpkm	log_2_(FC)	FDR
AY_1247_21697_26716	0.001	0.835	9.705632	0.000158
AY_602_157004_161798	0.355	6.095	4.101735	2.88 × 10^−8^
AY_20_711178_725997	0.17	2.07	3.606024	1.85 × 10^−^^7^
AY_34_1108397_1109641	110.745	934.43	3.076845	2.57 × 10^−^^9^
AY_1054_82728_85933	0.43	3.23	2.909126	0.016659
AY_34_288108_295647	0.625	3.52	2.493647	0.005137
AY_1024_125454_134463	1.165	0.575	−1.0187	1.49 × 10^−^^7^
AY_874_140748_143694	0.9	0.37	−1.2824	0.001709
AY_261_133645_148170	1	0.38	−1.39593	1.94 × 10^−^^11^
AY_545_152779_164768	12.995	4.155	−1.64504	1.65 × 10^−^^17^
AY_225_415711_427236	0.305	0.08	−1.93074	8.78 × 10^−^^5^
AY_7_943452_945369	0.71	0.001	−9.47168	0.001699

**Table 3:** DEGs that are potentially *cis*-regulated by DELs during ApNPV infection.

LTR ID	Gene ID	Gene Name	log_2_(FC)
AY_20_711178_725997	evm.TU.AY_20.26	probable E3 ubiquitin-protein ligase sinah [Papilio xuthus]	2.269461
AY_225_415711_427236	evm.TU.AY_225.12	facilitated trehalose transporter Tret1-like [Bombyx mori]	−2.0158
AY_225_415711_427236	XLOC_008622	lysine-specific demethylase lid [Papilio xuthus]	11.34707
AY_225_415711_427236	evm.TU.AY_225.14	lysine-specific demethylase lid isoform X2 [Bombyx mori]	3.464566
AY_225_415711_427236	XLOC_008623	--	11.94398
AY_261_133645_148170	evm.TU.AY_261.15	glucose dehydrogenase [FAD, quinone]-like [Bombyx mori]	−1.71236
AY_261_133645_148170	evm.TU.AY_261.19	protein singed [Bombyx mori]	1.400687
AY_261_133645_148170	evm.TU.AY_261.18	epsin-2 [Amyelois transitella]	1.159083
AY_34_288108_295647	evm.TU.AY_34.24	endonuclease and reverse transcriptase-like protein [Bombyx mori]	1.318608
AY_34_1108397_1109641	evm.TU.AY_34.65	protein transport protein Sec61 subunit alpha isoform 2 [Papilio machaon]	−1.35623
AY_34_1108397_1109641	evm.TU.AY_34.55	tyrosine-protein kinase CSK isoform X1 [Bombyx mori]	2.137612
AY_34_1108397_1109641	evm.TU.AY_34.57	DET1 homolog [Amyelois transitella]	1.614551
AY_34_1108397_1109641	evm.TU.AY_34.58	polyubiquitin-B [Drosophila bipectinata]	2.66362
AY_34_1108397_1109641	evm.TU.AY_34.63	midnolin [Papilio machaon]	1.787524
AY_545_152779_164768	XLOC_019487	protein split ends isoform X1 [Amyelois transitella]	5.019194
AY_545_152779_164768	XLOC_019488	--	3.989946
AY_545_152779_164768	XLOC_019489	protein split ends isoform X1 [Amyelois transitella]	4.9572955
AY_545_152779_164768	evm.TU.AY_545.6	ataxin-2-like protein [Bombyx mori]	3.027068
AY_545_152779_164768	evm.TU.AY_545.3	malate dehydrogenase, mitochondrial [Amyelois transitella]	−1.42245
AY_545_152779_164768	evm.TU.AY_545.1	nuclear receptor corepressor 1 [Bombyx mori]	4.495695
AY_874_140748_143694	evm.TU.AY_874.5	reverse transcriptase [Danaus plexippus]	9.748193
AY_874_140748_143694	evm.TU.AY_874.3	zinc finger protein OZF-like [Papilio xuthus]	2.395929
AY_874_140748_143694	evm.TU.AY_874.4	zinc finger protein 431-like [Bombyx mori]	2.895531

--: No gene name

## Supplementary Materials

The following are available online at https://www.mdpi.com/1999-4915/11/5/421/s1, Table S1: Properties of identified AyLTRs. Table S2: GO and KEGG pathway analysis of neighboring host genes (potential cis-targets) of “Stream” AyLTRs. Table S3: Expression data of LTR-retrotransposon in ApNPV-infected *A. pernyi* larval midgut samples and their uninfected controls.
